# Extracting the Infrared Permittivity of SiO_2_ Substrates Locally by Near-Field Imaging of Phonon Polaritons in a van der Waals Crystal

**DOI:** 10.3390/nano11010120

**Published:** 2021-01-07

**Authors:** Patricia Aguilar-Merino, Gonzalo Álvarez-Pérez, Javier Taboada-Gutiérrez, Jiahua Duan, Iván Prieto, Luis Manuel Álvarez-Prado, Alexey Y. Nikitin, Javier Martín-Sánchez, Pablo Alonso-González

**Affiliations:** 1 Department of Physics, University of Oviedo, 33006 Oviedo, Spain; UO258951@uniovi.es (P.A.-M.); gonzaloalvarez@uniovi.es (G.Á.-P.); taboadajavier@uniovi.es (J.T.-G.); duanjiahua@uniovi.es (J.D.); lmap@uniovi.es (L.M.Á.-P.); 2 Center of Research on Nanomaterials and Nanotechnology, CINN (CSIC-Universidad de Oviedo), 33940 El Entrego, Spain; 3 Institute of Science and Technology Austria, 3400 Klosterneuburg, Austria; i.prieto@me.com; 4 Donostia International Physics Center (DIPC), 20018 Donostia-San Sebastián, Spain; alexey@dipc.org; 5 IKERBASQUE, Basque Foundation for Science, 48009 Bilbao, Spain

**Keywords:** s-SNOM, phonon polaritons, van der Waals materials, infrared permittivity

## Abstract

Layered materials in which individual atomic layers are bonded by weak van der Waals forces (vdW materials) constitute one of the most prominent platforms for materials research. Particularly, polar vdW crystals, such as hexagonal boron nitride (h-BN), alpha-molybdenum trioxide (α-MoO_3_) or alpha-vanadium pentoxide (α-V_2_O_5_), have received significant attention in nano-optics, since they support phonon polaritons (PhPs)―light coupled to lattice vibrations― with strong electromagnetic confinement and low optical losses. Recently, correlative far- and near-field studies of α-MoO_3_ have been demonstrated as an effective strategy to accurately extract the permittivity of this material. Here, we use this accurately characterized and low-loss polaritonic material to sense its local dielectric environment, namely silica (SiO_2_), one of the most widespread substrates in nanotechnology. By studying the propagation of PhPs on α-MoO_3_ flakes with different thicknesses laying on SiO_2_ substrates via near-field microscopy (s-SNOM), we extract locally the infrared permittivity of SiO_2_. Our work reveals PhPs nanoimaging as a versatile method for the quantitative characterization of the local optical properties of dielectric substrates, crucial for understanding and predicting the response of nanomaterials and for the future scalability of integrated nanophotonic devices.

## 1. Introduction

The development of advanced nanophotonic devices relies heavily on the accurate characterization of the optical properties of nanomaterials. At the same time, to accurately predict the optical response of nanomaterials, it is essential to obtain a precise dielectric function model for them. A useful resource to sense such optical properties is provided by strongly confined polaritons—hybrid light–matter excitations. Particularly, the properties of phonon polaritons (PhPs) in polar vdW materials [1,2], such as h-BN [3,4,5], α-MoO_3_ [6,7,8,9,10,11,12], or α-V_2_O_5_, [13], are extremely sensitive to their dielectric environment, especially to the substrate on which the crystal is placed [14,15]. In this context, amorphous SiO_2_ substrates are among the most utilized for many applications in nanophotonics [1,2,3,4,5,6,7,8,9,10,11]. However, there are significant discrepancies between most of the reported works throughout the literature for the estimation of the infrared (IR) dielectric permittivity of SiO_2_ [16,17,18,19,20]. Such discrepancies are mainly attributed to differences in the growth process of SiO_2_, which leads to oxides with non-controllable stoichiometry, impurities like intercalated aluminum atoms, random fluctuations in hydroxyl concentration, and variable mechanical stress at the SiO_2_/Si layer interface. On the other hand, the measurement of the IR dielectric permittivity has relied on traditional far-field techniques (diffraction-limited), such as ellipsometry or Fourier-transform infrared spectroscopy (FTIR), with a micrometer resolution in the best of cases. In contrast, by beating the diffraction limit of light, near-field optical microscopy (s-SNOM) exhibits instrumental advantages with respect to traditional techniques, such as extreme sensitivity to the optical losses of the material and nanometer spatial resolutions capable of determining the complex dielectric permittivity of nanomaterials with extraordinary accuracy [21,22,23].

In this work, we perform nanoimaging of PhPs in α-MoO_3_ flakes laying on SiO_2_ substrates to characterize the complex IR dielectric permittivity of SiO_2_ with nanometer spatial resolution. Specifically, the IR dielectric permittivity of SiO_2_ is obtained by fitting the experimental dispersion of PhPs in α-MoO_3_/SiO_2_ with that calculated by a transfer matrix method and analytical calculations which consider the recently reported IR permittivity of α-MoO_3_ [24]. The as-obtained dielectric permittivity of SiO_2_ is further corroborated by full-wave numerical simulations, which are in good agreement with our experimental results.

## 2. Materials and Methods

### 2.1. Sample Fabrication

Firstly, thin α-MoO_3_ crystals with thicknesses of several hundreds of nanometers are produced by thinning down commercial bulk materials (Alfa Aesar) upon mechanical exfoliation employing Nitto blue tape. Then, the as-produced flakes are transferred from the tape to a transparent polydimethylsiloxane (PDMS) stamp, on top of which they are inspected by optical microscopy in transmission mode. Selected flakes with homogeneous shapes and heights are finally peeled off from the PDMS stamp to SiO_2_/Si substrates (300 nm-thick amorphous SiO_2_ layer grown on (100)-Si from Sil’Tronix Silicon Technologies). During this process, the substrates are kept at a temperature of 200 °C to maximize the transfer yield of flakes. The flakes are localized on the substrate with respect to gold markers defined by optical lithography.

### 2.2. Fabrication of Gold Optical Nanoantennas

For efficient excitation of PhPs in α-MoO_3_/SiO_2_, we employ rod-like gold nanoantennas fabricated on top of the α-MoO_3_ flakes by electron beam lithography (100 kV). To do this, the substrates containing the α-MoO_3_ crystals are first coated with a PMMA resist layer. Upon exposure with the electron beam, the samples are treated with a conventional high-resolution developer (1:3 MIBK: IPA) followed by evaporation of a Cr(5 nm)/Au(30 nm) bilayer. The lift-off is performed by dipping the sample into a hot acetone bath at 60 °C for 10–15 min and a gentle rinse of IPA for 1 min, followed by a nitrogen gas drying. The dimensions of the obtained gold nanoantennas are 3 μm (length) × 50 nm (width) × 40 nm (height).

### 2.3. Scattering-Type Scanning Near-Field Optical Microscopy (s-SNOM)

Near-field optical characterization is performed by infrared nanoimaging using a commercially available scattering-type near-field optical microscope, s-SNOM, from Neaspec. The gold nanoantenna is illuminated by focusing the incoming IR light from a tunable CO_2_ laser, or a quantum cascade laser, at frequency $\omega_{0}$ and p-polarization with a parabolic mirror. A metallized (Pt-coated) atomic force microscope tip is used as a scattering near-field probe while it oscillates at the mechanical resonant frequency of the cantilever (around 270 kHz) with an amplitude of about 100 nm (tapping mode). The tip-scattered field together with part of the incident light impinging on a pseudo-heterodyne Michelson interferometer are collected into an IR detector. Demodulation of this interferometric signal at the nth harmonics of the tip oscillation frequency allows us to subtract the background signal, yielding the complex-valued near-field $\sigma_{n}=s_{n}e^{i\phi_{n}}$, where $s_{n}$ and $\phi_{n}$ are the near-field amplitude and phase, respectively. For the near-field measurements performed in this work, demodulation of the near-field signal was carried out at n=3. The near-field images are obtained by recording the near-field signals as a function of the lateral tip position over the sample.

### 2.4. Full-Wave Numerical Simulations

Full-wave numerical simulations of the near-field signal on the heterostructure studied in this work (consisting of a 225-nm-thick α-MoO_3_ biaxial slab placed on top of a SiO_2_ substrate) are obtained by finite element calculations using the commercial software Comsol Multiphysics. A vertically oriented electric point dipole above the surface of the α-MoO_3_ slab (at a height of 200 nm) was used for the excitation of PhPs. All images show the real part of the z-component of the electric field, ReEzx,y, above the substrate surface at a height of about 100 nm.

## 3. Results and Discussion

The dielectric permittivity of SiO_2_ in the mid-IR region (from 9 to 12 μm, or, equivalently from 1110 to 830 cm^−1^) can be inferred by analyzing the optical properties of propagating PhPs in α-MoO_3_/SiO_2_ heterostructures, as PhPs in α-MoO_3_ flakes are extremely sensitive to the dielectric environment, i.e., to the underlying SiO_2_ substrate. In particular, the wavelength of PhPs $\lambda_{p}$ (or, equivalently, the polaritonic wavevector $k_{p}=\frac{2\pi }{\lambda_{p}}$) in α-MoO_3_ slabs is strongly dependent on the dielectric permittivity of the superstrate (air, $\varepsilon_{1}=1$) and the substrate ($\varepsilon_{{\mathrm{SiO}}_{2}}$) as shown by the analytical dispersion in Equation (1) [25].

(1) $$k_{p}\left(\omega \right)=\frac{\rho }{d}\left[\arctan\left(\frac{\varepsilon_{1}\rho }{\varepsilon_{z}}\right)+\arctan\left(\frac{\varepsilon_{{\mathrm{SiO}}_{2}}\rho }{\varepsilon_{z}}\right)+\pi l\right],\;\;\;\;\;\;\;\;l=0,\;1,\;2\ldots$$

where *d* is the thickness of the α-MoO_3_ flake, $k_{p}$ is the polaritonic in-plane momentum, and thus $k_{p}$ is its modulus *(*$k_{p}^{2}=k_{px}^{2}+k_{py}^{2}$*)*, α is the angle between the [100] crystal direction of α-MoO_3_ and $k_{p}$, $\rho =i\sqrt{\varepsilon_{z}/\left(\varepsilon_{x}\cos^{2}\alpha +\varepsilon_{y}\sin^{2}\alpha \right)}$, $\varepsilon_{i}\;\left(i=x,y,z\right)$ are the components of the anisotropic dielectric permittivity of α-MoO_3_, and l is the mode index. On the other hand, the dielectric permittivity of SiO_2_,$\;\varepsilon_{{\mathrm{SiO}}_{2}}$, can be expressed by the Lorentz model [26,27] with three coupled oscillators:

(2) $$\varepsilon_{{\mathrm{SiO}}_{2}}\left(\omega \right)=\varepsilon_{\infty }\prod_{j=1}^{3}\frac{\omega_{LO,j}^{2}-\omega^{2}-i\gamma_{j}\omega }{\omega_{TO,j}^{2}-\omega^{2}-i\gamma_{j}\omega },$$

where $\varepsilon_{\infty }$ represents the high-frequency dielectric constant, $\omega_{TO,\;j}\;$ and $\omega_{LO,\;j}$ refer to the transverse (TO) and longitudinal (LO) optical phonon frequencies, respectively, and $\gamma_{j}$ represents the damping factor of the Lorentzian line shapes. These parameters are, therefore, the free parameters when adjusting the dielectric permittivity. Finally, j is the subscript denoting the different phonon LO-TO pairs. It is worth mentioning that s-SNOM polariton imaging is extremely sensitive to both phonon frequencies and the phonon damping of the substrate. More specifically, the PhPs wavelength is very sensitive to TO and LO phonon frequency variations, while the measured propagation length is dictated by damping. Therefore, the abovementioned fitting parameters can be obtained with high accuracy by extracting the PhP wavelength and propagation length at different illuminating frequencies from experimental near-field images.

**Figure 1:**
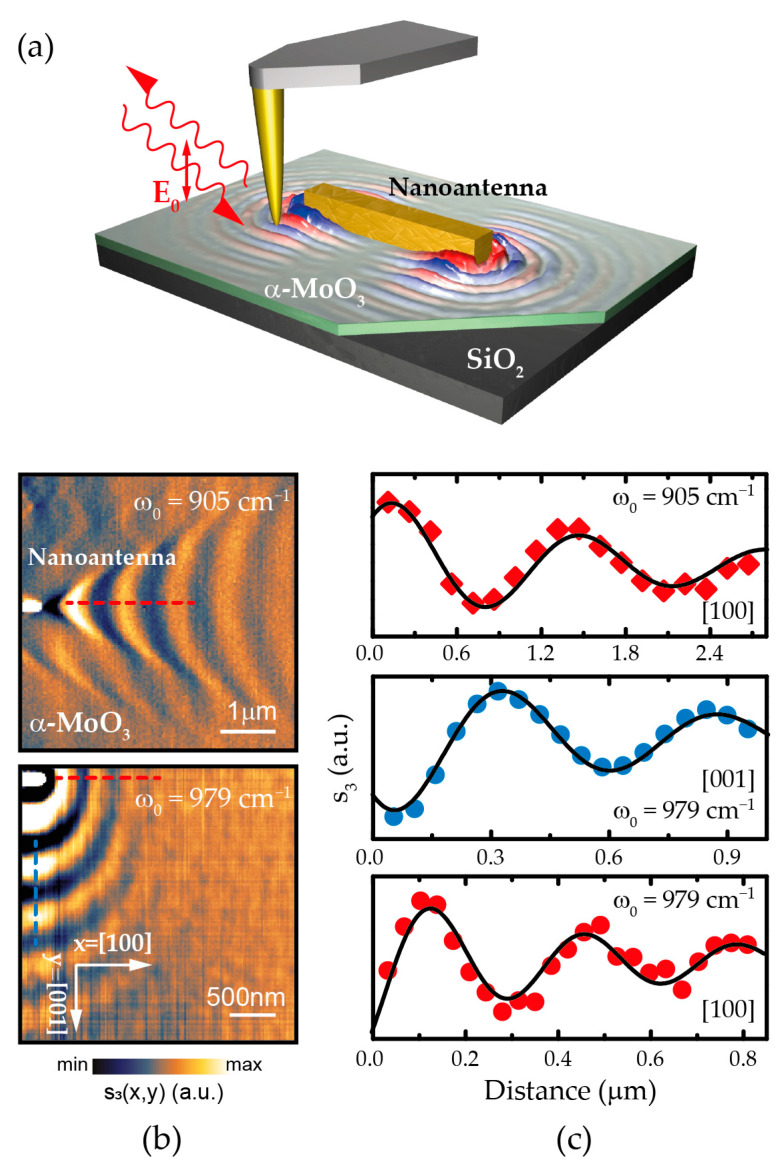
(**a**) Schematics of the experimental setup employed to image propagating PhPs in a α-MoO_3_ flake transferred on a SiO_2_ substrate. A gold rod-like nanoantenna is used to confine the incident infrared light with p-polarized field *E*_0_, which allows the excitation of PhPs. The near-field signal on the surface of the slab is scattered by a metallic tip and detected by a distant detector. (**b**) Experimental near-field amplitude images (*s*_3_(*x*,*y*)) launched by the nanoantenna in a 225-nm-thick (see Appendix A) α-MoO_3_ flake at illuminating frequencies ω_0_ = 905 cm^−1^ (hyperbolic regime, top panel) and *ω*_0_ = 979 cm^−1^ (elliptic regime, bottom panel). (**c**) Experimental profiles (dots) taken along the [100] and [001] directions denoted by dashed lines in (**b**). The fit to the experimental data is depicted with a black solid line.

To experimentally visualize propagating PhPs, we perform near-field s-SNOM measurements on α-MoO_3_ flakes, in which rod-like gold nanoantennas fabricated on top of the sample are used to excite PhPs, at an illuminating frequency *ω*_0_ and with p-polarized light, as shown in Figure 1a. The near-field signal is measured at each position over the surface of the flake by collecting the scattered near-field signal by a metallized AFM tip. We note that the strong anisotropy of the α-MoO_3_ crystal yields two IR “reststrahlen bands” (RB, spectral regions defined between the TO and LO phonon frequencies, and in which the dielectric permittivity is negative, therefore enabling the existence of PhPs), in the frequency range between 820 cm^−1^ and 1010 cm^−1^. The in-plane propagation of PhPs is strongly anisotropic with characteristic elliptic or hyperbolic dispersion depending on the illuminating frequency [6,7]. Typically, those bands are labeled RB2 (hyperbolic, 821–963 cm^−1^) [7,24] and RB3 (elliptical, 957–1007 cm^−1^) [7,24]. Figure 1b shows the s-SNOM near-field amplitude images ($s_{3}$) obtained at representative frequencies residing in the hyperbolic (*ω*_0_ = 905 cm^−1^) and elliptic (*ω*_0_ = 979 cm^−1^) regimes, respectively. In the former, highly directional hyperbolic PhP propagation is observed with concave wavefronts centered along the [100] crystal direction in α-MoO_3_. Note that no discernible PhP propagation is observed along the [001] direction. On the contrary, elliptic-shaped propagation of PhPs is observed in the RB3 with a larger polariton wavelength $\lambda_{p}$ (smaller wavevector $k_{p}$) along the [001] crystal direction with respect to the [100] direction. The PhP propagation length $L_{p}$ and wavelength $\lambda_{p}$ are obtained by fitting Equation (3) to the experimental profiles drawn along the crystal directions [100] and [001] on the near-field monochromatic images, as shown in Figure 1c. Note that the decay of polaritons away from an edge (in this case, the rod) is due to a combination of damping (Imkp>0) and geometric spreading [28].

(3) $$\xi_{\mathrm{opt}}\left(x\right)=\xi_{0}+A\frac{e^{-\frac{2x}{L_{p}}\;}\sin\left(4\pi \frac{x-x_{A}}{\lambda_{p}}\right)}{\sqrt{x}}+B\frac{e^{-\frac{x}{L_{p}}\;}\sin\left(2\pi \frac{x-x_{B}}{\lambda_{p}}\right)}{x^{a}},\;\;\;\;\;A,B,L_{p},\lambda_{p},a>0,$$

where $\;A,B,a,x_{A},x_{B}$ are fitting constants. Usually, polaritons are excited by the tip, travel along the flake, and get reflected at sample discontinuities. As such, the first term in the summation represents the polaritonic field returning to the tip of the s-SNOM for a damped wave reflected from a discontinuity (typically the edge of the sample), with the PhPs thus traveling a distance 2x. The second term arises because PhPs are not only generated at the tip apex, but also at the edge of the slab, traveling then a distance x to the tip. However, in our configuration, we consider the tip acting only as detector rather than a launcher as the Au nanoantenna launches PhPs more efficiently than reflect them. Thus, only periodic $\lambda_{p}$ waves with circular geometry decay (note that hyperbolic waves decay geometrically as circular waves [6,24]) are present and, therefore, we set A=0 and a=0.5 to properly fit this experimental setting. The fact of polaritons being launched by Au nanoantennas simplifies the analysis, as only the field corresponding to antenna-launched polaritons needs to be fitted, rather than both antenna- and tip-launched polaritons. From the experimental point of view, this configuration enables direct visualization of the polaritonic wavefronts [29].

The free parameters $\omega_{LO,j}$, $\omega_{TO,j}\;$ and $\gamma_{j}$ are therefore adjusted to reproduce the experimentally obtained PhPs wavelength $\lambda_{p},\;i.e.,\;\mathrm{polaritonic}\;\mathrm{wavevector}\;k_{p},$ and propagation length $L_{p}$ through Equations (1) and (2) based on an iterative procedure (Figure 1c). Particularly, within RB2 (*ω*_0_ = 905 cm^−1^), we experimentally observe fringes along the [100] crystal direction (Figure 1b, top panel), which indicate the excitation of in-plane hyperbolic PhPs in α-MoO_3_, consistent with what has been previously reported [6,7]. In turn, within RB3 (*ω*_0_ = 979 cm^−1^), we observe in the experimental image (Figure 1b, bottom panel) fringes along both in-plane directions. The experimental PhPs wavelengths and propagation lengths along the [100] and [001] crystal directions are shown in Table 1.

**Figure 2:**
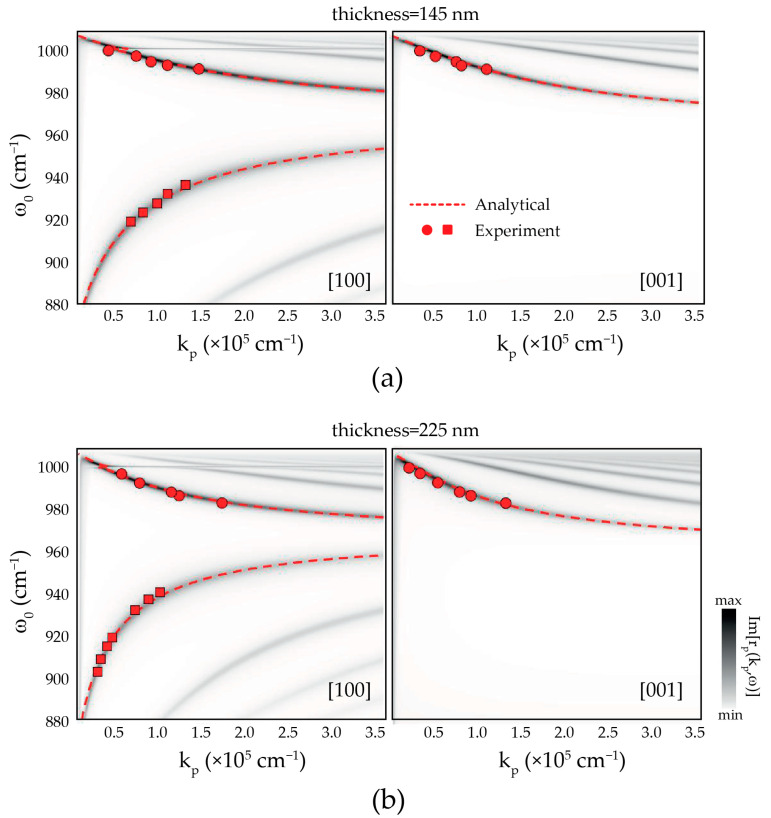
Dispersion of PhPs on thin α-MoO_3_ flakes on top of SiO_2_ substrates. Transfer-matrix calculations (false color plot) and analytical polaritonic dispersions (dashed red lines) of PhPs propagating in an α-MoO_3_ slab with thickness (**a**) 145 nm and (**b**) 225 nm, along the [100] (**left**) and [001] (**right**) crystal directions of α-MoO_3_. Red symbols represent the experimental data obtained from monochromatic s-SNOM near-field images.

These fitting parameters are further fine-tuned by comparing the experimental polaritonic wavevector, obtained at different illumination frequencies *ω*_0_ from about 870 cm^−1^ to 1000 cm^−1^ and different α-MoO_3_ flake thicknesses (red symbols), to the polaritonic dispersion, obtained from both analytical calculations (dashed lines) [25] and transfer-matrix calculations (dark maxima in the color plots) [30] (Figure 2). In the latter, the polaritonic dispersion corresponds to the divergences of the imaginary part of the Fresnel reflection coefficient, Imrp, of the structure at complex $k_{p}$. An excellent agreement between the experiment and the transfer matrix and analytical calculations is obtained.

We emphasize that, as shown in Equation (1), besides the dependence of the polaritonic wavelength on the dielectric permittivity of the slab and the surrounding media, and particularly the SiO_2_ substrate, it also depends inversely on the flake’s thickness. The later adding an additional tuning knob for a finer estimation of the dielectric permittivity. Hence, the successful fitting of the polaritonic response for different thicknesses in α-MoO_3_ slabs reinforces our claims of a robust quantitative modelling of the IR SiO_2_ dielectric permittivity [3,6].

**Figure 3:**
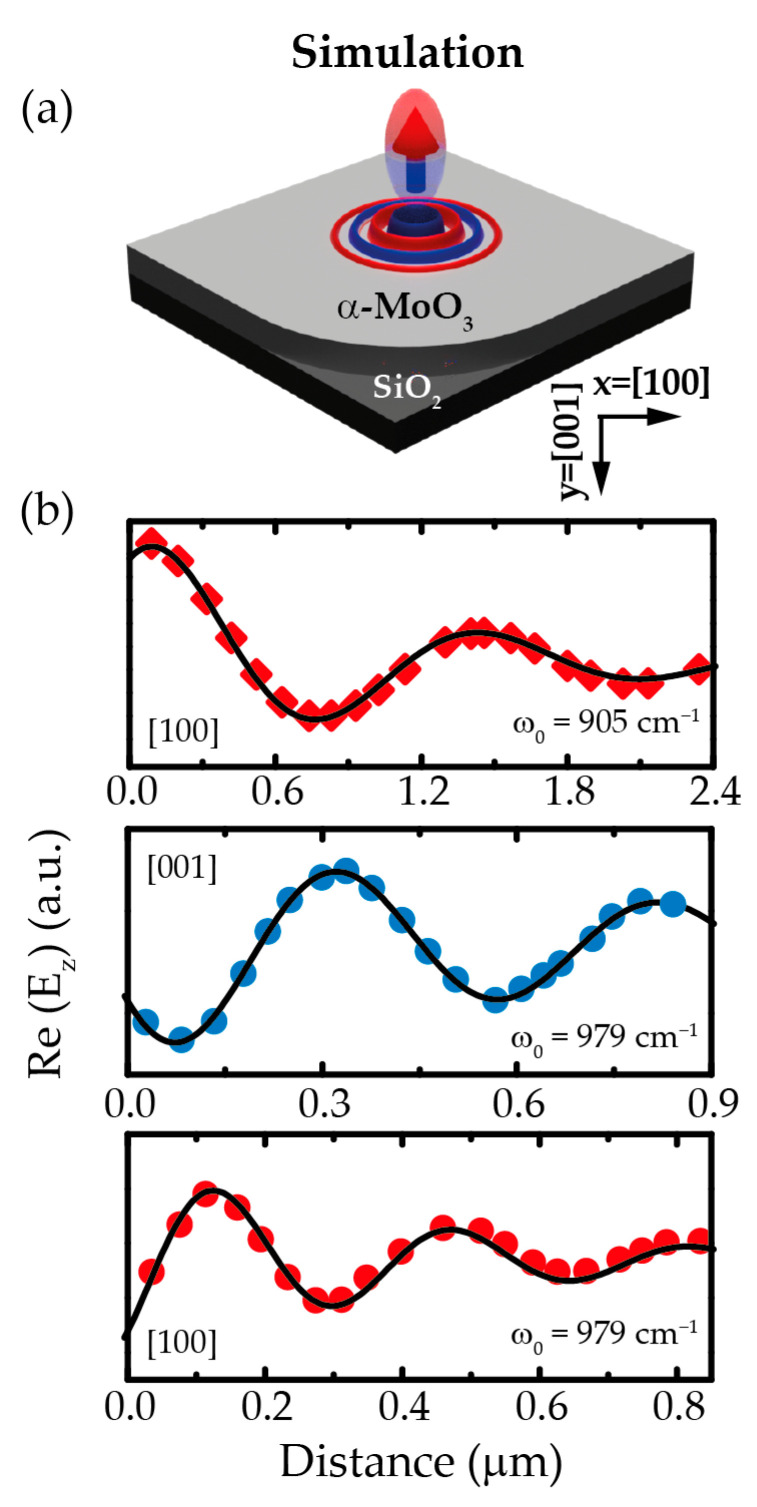
(**a**) Schematics of a 225-nm-thick α-MoO_3_ slab on a SiO_2_ substrate in which propagating PhPs are depicted by elliptical fringes (represented in red and blue). A vertically oriented electric point dipole placed on top of the structure provides concentrated electric fields that allow for launching PhPs at an incident wavelength $\omega_{0}=$ 979 cm^−1^ (elliptic regime). (**b**) Simulated electric field profiles (dots), Re(E_z_), along the [100] and [001] crystal directions of α-MoO_3_ for an incident frequency $\omega_{0}=$ 905 cm^−1^ (hyperbolic regime) and $\omega_{0}=$ 979 cm^−1^ (elliptic regime). The fit to the simulated profiles are depicted with a black solid line.

To further corroborate the extracted SiO_2_ dielectric permittivity and account for the experimental results with our dielectric function, we run full-wave numerical simulations of PhPs (launched by an electric point dipole) in a 225-nm-thick α-MoO_3_ flake laying on a SiO_2_ substrate characterized by the extracted permittivity at representative frequencies in the hyperbolic (*ω*_0_ = 905 cm^−1^) and elliptic (*ω*_0_ = 979 cm^−1^) regimes, respectively (Figure 3). The corresponding simulated profiles of PhPs propagating along the [100] and [001] crystal directions are then fitted following Equation (3). Note that to fit these profiles, we set A = 0 and a = 0.5 in Equation (3), such as for the experimental profiles since PhPs are launched by a point dipole (in analogy to the nanoantennas in the experiments).

From the fit to the simulated profiles (Figure 3b), we extract the values for the polaritonic wavelengths (${\hat{\lambda }}_{p}$), as shown in Table 2. These values are in good agreement with the experiment, demonstrating the validity of our model. Finally, we adjust the damping factors, $\gamma_{j}$, of the two SiO_2_ phonons close to RB2 and RB3 (phonon j=2 with *ω_TO_* ≈ 800 cm^−1^ and phonon j=3 with *ω_TO_* ≈ 1045 cm^−1^, respectively), by fitting the PhP propagation lengths ${\hat{L}}_{p}$ in the simulated profiles to those in the experiment. Table 2 shows the obtained values. These values differ slightly from the experiment. We note that the best fit to the experimental profiles is obtained for a relatively weak phonon damping of $\gamma_{2}=\gamma_{3}=10$ cm^−1^, in agreement with prior works on near-field imaging of polaritons [31]. This surprising result, as well as the differences obtained between the simulated and the experimental propagation lengths, can be explained by a poor adhesion of the α-MoO_3_ flake to the SiO_2_ substrate, which may lead to the formation of air gaps at the interface between them. The precise characterization of such air gaps and their effect on the propagation of PhPs and on the effective permittivity of the substrate provides an interesting avenue for future work.

**Figure 4:**
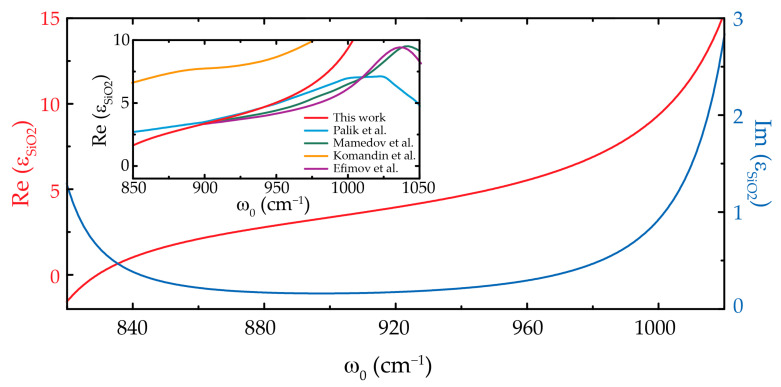
Experimental IR complex dielectric function of SiO_2_. The real and imaginary parts are depicted in blue and red, respectively. The inset shows a comparison of the extracted permittivity with others obtained from far-field measurements.

The resulting fitting parameters for the permittivity of SiO_2_, according to Equation (2), are shown in Table 3 and the calculated IR dielectric permittivity is plotted in Figure 4 together with those reported by Komandin et al. [17], Palik [18], Mamedov et al. [19] and Efimov [20].

## 4. Conclusions

In summary, by performing near-field polariton nanoimaging we have extracted locally the complex dielectric function of SiO_2_ at IR frequencies. Its robustness is demonstrated by reproducing numerically different experimental measurements in slabs of different thicknesses. We achieved this by combining the virtues of monochromatic near-field imaging of polaritons, such as sensitivity to phonon damping and LO phonon energies, with advanced theoretical and numerical approaches. The extension of this procedure to broadband sources, such as those used in Fourier transform infrared nanospectroscopy [21,22,23], holds great promises to extracting dielectric functions of isotropic and anisotropic nanomaterials.

Our work provides an alternative to predict the dielectric function of nanophotonic materials, and therefore to extract the local optical response of 2D, nano- and low-dimensional materials. As many nanophotonic and optoelectronic devices are based on SiO_2_, we anticipate future and optimized experiments using the extracted permittivity, as well as correlative far- and near-field characterization studies [24]. Furthermore, as the permittivity of the substrate plays an important role in the propagation of polaritons, our near-field procedure to extract the substrate local permittivity facilitates the development of planar nanophotonic technologies, especially based on the exotic phenomena that recent reports of in-plane hyperbolicity have demonstrated in low-loss natural crystals. For instance, the use of local changes in the dielectric environment or the realization of advanced concepts for reconfigurable planar meta-optics [32,33].

## Figures and Tables

**Table 1:** Wavelengths ($\lambda_{p}$) and propagation lengths ($L_{p}$) obtained from the experimental profiles shown in Figure 1.

$\omega_{0\;}\left(cm^{-1}\right)$	*Crystal Axis*	$\lambda_{p}(\mathrm{nm})$	$L_{p}(\mu m)$
905	100	1330±15	2.79±0.15
979	100	330±15	1.39±0.15
979	001	545±15	1.30±0.15

**Table 2:** Wavelengths (${\hat{\lambda }}_{p}$) and propagation lengths (${\hat{L}}_{p}$) obtained from the simulated profiles shown in Figure 3.

$\omega_{0}\;\left(cm^{-1}\right)$	*Crystal Axis*	${\hat{\lambda }}_{p}(\mathrm{nm})$	${\hat{L}}_{p}(\mu m)$
905	100	1330±15	1.37±0.15
979	100	345±15	0.510±0.15
979	001	495±15	1.29±0.15

**Table 3:** Parameters for the calculated IR complex dielectric function of SiO_2_.

$\varepsilon_{\infty }$	j(Phonon Index)	$\omega_{TO,j}\;\left(cm^{-1}\right)$	$\omega_{LO,j}\;\left(cm^{-1}\right)$	$\gamma_{j}\;\left(cm^{-1}\right)$
2	1	450	505	51
	2	800	830	10
	3	1045	1240	10

**Figure A1:**
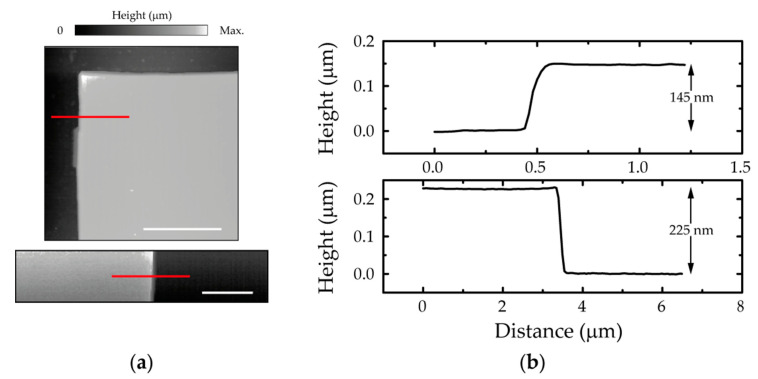
(**a**) AFM images of the α-MoO_3_ flakes employed in this work. The scale bar is 2 µm. (**b**) Height profiles taken along the red solid lines. The thicknesses of the flakes are 145 nm (**top** panel) and 225 nm (**bottom** panel) as measured from the profiles with respect to the SiO_2_ substrate.

## Data Availability

The data presented in this study are available on request from the corresponding authors.

## Appendix A

Figure A1a shows the atomic force microscopy (AFM) images of the two α-MoO_3_ flakes measured by near-field imaging in this work. As can be seen in the AFM images, the edges of the flakes are regular and the profiles taken along the red solid line (Figure A1b) show that the flakes are atomically flat along all crystal directions. The thicknesses of the flakes are 145 nm and 225 nm.
